# Endocrine therapy and thyroid function in early luminal breast cancer: a controlled longitudinal retrospective analysis

**DOI:** 10.3389/fonc.2026.1821253

**Published:** 2026-06-18

**Authors:** Zengwei Li, Tingfei Song, Yu Zhang, Yonghui Wang, Hui Sun

**Affiliations:** 1 School of Clinical Medicine, Shandong Second Medical University, Weifang, Shandong, China; 2 Department of Thyroid Surgery, Weifang People’s Hospital, Weifang, Shandong, China; 3 Department of Breast Surgery, Weifang People’s Hospital, Weifang, Shandong, China

**Keywords:** breast cancer, endocrine therapy, tamoxifen, aromatase inhibitors, thyroid function

## Abstract

**Background:**

Endocrine therapy is a standard treatment strategy for hormone receptor-positive breast cancer, but the long-term impact on thyroid function remains unclear. This study evaluates changes in thyroid function among patients with early luminal breast cancer receiving adjuvant endocrine therapy compared to those with benign breast disease (BBD).

**Methods:**

This retrospective longitudinal cohort study included 53 patients with early luminal breast cancer (BC) who received adjuvant tamoxifen (TAM) or aromatase inhibitors (AIs), and 21 BBD controls. All participants had no history of thyroid disease, chemotherapy, or radiotherapy. Thyroid function parameters (TSH, FT3, FT4) were measured at baseline, 1 year, and 3 years post-surgery. Generalized linear mixed models (GLMM) were used to compare longitudinal changes between groups.

**Results:**

After 3 years of endocrine therapy, the BC group showed a significant increase in TSH (from 1.92 to 3.06 μIU/mL) and decreases in FT3 (from 4.90 to 4.14 pmol/L) and FT4 (from 18.35 to 15.26 pmol/L), whereas the BBD group showed no statistically significant changes over time. Significant group-by-time interactions were found for TSH (P = 0.018), FT3 (P = 0.016), and FT4 (P < 0.001). In subgroup analysis comparing TAM and AIs, a significant interaction was observed for FT4 (P = 0.022), indicating a greater decline in FT4 with TAM, while no significant differences were found for TSH (P = 0.508) or FT3 (P = 0.204). In subgroup analysis by tumor grade, no significant time-by-grade interactions were observed for TSH (P = 0.210), FT3 (P = 0.186), or FT4 (P = 0.073).

**Conclusions:**

Adjuvant endocrine therapy in early luminal breast cancer was associated with statistically significant alterations in thyroid function over 3 years, characterized by increased TSH and decreased FT3 and FT4. These alterations did not differ substantially by tumor grade. However, the small sample size and the complete collinearity between treatment type and menopausal status preclude drawing independent conclusions regarding the differential effects of TAM versus AIs. Further prospective studies are warranted to validate these observations and to explore their potential clinical relevance.

## Introduction

Breast Cancer (BC) is the most common malignancy among women worldwide. While advances in diagnosis and treatment have significantly improved survival rates, growing attention has been directed toward the long-term side effects of these therapies (1). Hormone receptor-positive (HR+) breast cancer is the most prevalent subtype, accounting for approximately 70-80% of all breast cancer cases. In a large U.S. population-based study using SEER data, the HR+/HER2- subtype alone comprised 72.7% of cases with known hormone receptor and HER2 status (2). For these patients, adjuvant endocrine therapy is a cornerstone of treatment to reduce the risk of recurrence and improve long-term prognosis (3, 4). Tamoxifen (TAM), a selective estrogen receptor modulator (SERM), is commonly used in premenopausal and perimenopausal women, while aromatase inhibitors (AIs) are typically reserved for postmenopausal women, though both may be used in select clinical scenarios according to menopausal status (5, 6). Despite their established efficacy, these agents may inadvertently affect other endocrine organs by suppressing estrogen signaling, and their impact on thyroid function remains a subject of ongoing debate.

The thyroid and mammary glands are physiologically and pathologically interconnected. Estrogen can influence thyroid function indirectly by modulating thyroxine-binding globulin (TBG) levels and directly by acting on estrogen receptors within thyroid tissue (7). Moreover, thyroid hormones themselves exhibit estrogen-like activity and can regulate breast cell proliferation and differentiation (8). Thyroid hormone receptors, which are widely expressed in breast cancer, interact with estrogen receptors and other cancer-related pathways, potentially eliciting diverse biological effects (9). Therefore, it is theoretically plausible that anti-estrogen endocrine therapy could disrupt this complex interplay, indirectly affecting thyroid hormone synthesis, release, or metabolism, and consequently leading to thyroid dysfunction.

Existing studies on the effects of endocrine therapy for breast cancer on thyroid function have reported inconsistent findings (10–13). However, many of these studies are confounded by the inclusion of patients who received chemotherapy or radiotherapy, making it difficult to discern whether thyroid function alterations are attributable to endocrine therapy itself, multimodal treatment, or the underlying tumor biology (14). Furthermore, the lack of a benign breast disease (BBD) control group precludes the determination of whether observed thyroid abnormalities are inherent to breast cancer itself. Additionally, most published studies are limited to short-term follow-up, leaving the long-term impact on thyroid function inadequately characterized.

To address these gaps, we conducted a retrospective longitudinal cohort study that included only postoperative patients with early-stage luminal breast cancer who received endocrine therapy at our institution between 2016 and 2020, along with a control group of postoperative patients with BBD. The primary objective was to compare postoperative thyroid function trajectories between patients treated with AIs or TAM and the benign control group. Secondary analyses examined thyroid function differences between the AIs and TAM subgroups, with the goal of providing clinically relevant evidence to inform endocrine therapy management and long-term post-operative thyroid function monitoring in this patient population.

## Materials and methods

### Patients

Between January 2016 and December 2020, a total of 2,366 patients underwent breast cancer surgery at the Department of Breast Surgery, Weifang People’s Hospital. We retrospectively reviewed clinical data, imaging, and pathology reports. Patients were included if they met the following criteria: pathologically confirmed invasive breast cancer; immunohistochemical subtyping consistent with Luminal A (hormone receptor-positive, HER2-negative, Ki-67 < 14%) or Luminal B (hormone receptor-positive, HER2-positive or negative, Ki-67 ≥ 14% or low PR expression); no prior chemotherapy or radiotherapy; initiation of continuous adjuvant endocrine therapy with either an AIs or TAM for at least 12 months, without agent switching, treatment interruption, or a gap exceeding three months; and complete thyroid function test (TSH, FT3, FT4) records available both before endocrine therapy initiation and during follow-up. Exclusion criteria comprised a history of thyroid disease or use of thyroid-affecting medication, prior neck radiation, other significant endocrine disorders (including pituitary disorders affecting central thyroid regulation), prior or concomitant malignancy, and evidence of local recurrence or distant metastasis. Non-luminal subtypes were excluded because adjuvant endocrine therapy is not standard for them, and their inclusion would introduce biological heterogeneity that could confound the assessment.

**Figure 1:**
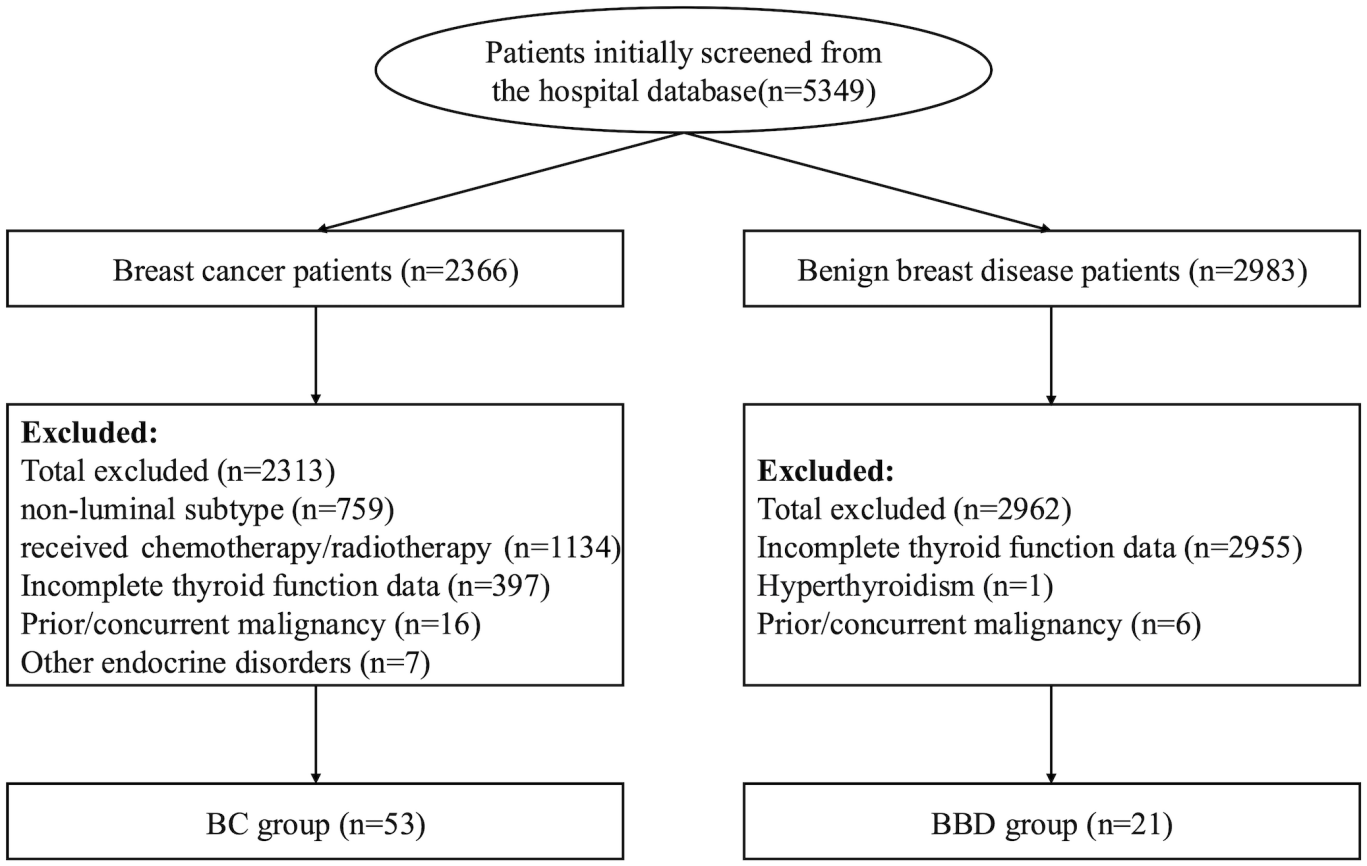
Flow diagram of patient selection.

After applying the criteria, the final BC group comprised 53 patients with early-stage, node-negative luminal disease (31 Luminal A, 22 Luminal B), none of whom received chemotherapy or radiotherapy. For the control group, we screened patients who underwent surgery for BBD during the same period using the identical exclusion criteria. Of 22 initially eligible patients, one was later excluded because of hyperthyroidism treated with propylthiouracil, leaving 21 BBD controls. The selection process is shown in **Figure 1**. The study was approved by the Institutional Review Board of Weifang People’s Hospital, which waived the requirement for written informed consent owing to the retrospective nature of the study.

### Data analysis

Thyroid function tests were performed at baseline (before initiating endocrine therapy) and at 1-year and 3-year follow-up visits. Serum levels of FT4, FT3, TSH, TgAb, and TPOAb were measured by electrochemiluminescence immunoassay (ECLIA) on a Roche Cobas e601 analyzer (Roche Diagnostics, Mannheim, Germany). Reference ranges were as follows: FT4, 10.44–24.38 pmol/L; FT3, 2.77–6.31 pmol/L; TSH, 0.380–4.340 μIU/mL; TgAb, 0–4.5 IU/mL; TPOAb, 0–60 IU/mL. Overt hyperthyroidism was defined as suppressed TSH with elevated FT4; overt hypothyroidism as elevated TSH with decreased FT4. Subclinical hypothyroidism was defined as elevated TSH with normal FT4 concentrations. The presence of autoantibodies (TgAb or TPOAb) indicated autoimmune thyroid disease.

### Statistical analysis

Generalized linear mixed models (GLMM) with a Gamma distribution and log link function were used to compare longitudinal trajectories of thyroid function parameters between groups, accounting for the positive skewness of TSH, FT3, and FT4. Fixed effects included time (categorical: baseline, 1 year, 3 years), group (BC vs. BBD for the primary analysis; TAM vs. AI for the treatment subgroup analysis; histological grade 2–3 vs. grade 1 for the grade subgroup analysis), and the time **×** group interaction. Sensitivity analyses were performed by separately adding the following covariates to the primary GLMM: age, BMI, menopausal status, baseline TSH level, TgAb positivity, TPOAb positivity, and the presence of diffuse heterogeneous echotexture on thyroid ultrasound. Given the limited sample size, random effects were restricted to a random intercept for each subject to ensure model convergence. The exponentiated coefficient (exp(β)) for the time × group interaction represented the ratio of geometric mean changes from baseline to 3 years between groups. Reference categories were the BBD group, the AIs group, and histological grade 1 for the respective comparisons. The significance of the time-by-group interaction was assessed using likelihood ratio test (LRT) comparing models with and without the interaction term.

Continuous variables were assessed for normality using the Shapiro-Wilk test. Normally distributed variables were expressed as mean ± standard deviation and compared using the independent-samples t-test; non-normally distributed variables were expressed as median (interquartile range) and compared using the Mann–Whitney U test. Categorical variables were presented as frequencies and percentages, and group comparisons were performed using Fisher’s exact test. All tests were two-sided, and P < 0.05 was considered statistically significant.

## Results

We analyzed a total of 74 subjects, comprising 53 patients with early-stage luminal breast cancer and 21 BBD controls. Their baseline demographic and clinical characteristics are summarized in **Table 1**. In the BC group, 31 cases (58.5%) were Luminal A and 22 cases (41.5%) were Luminal B. Histological types were as follows: 47 patients (88.7%) had invasive ductal carcinoma (IDC), 1 patient (1.9%) had invasive lobular carcinoma (ILC), and 5 patients (9.4%) had other special types (**Table 1**). Among the 53 BC patients, 29 had grade I malignancy and 24 had grade II/III. All patients had node-negative, early-stage disease. Per local guidelines, patients with grade I disease did not receive adjuvant chemotherapy or radiotherapy. Among the 24 patients with grade II/III disease, all declined chemotherapy and radiotherapy; five of them, along with their families, cited advanced age as the primary reason, while the remaining 19 declined for other personal considerations. Of these, 21 (39.6%) received TAM and 32 (60.4%) received AIs. Menopausal status was distributed as follows: in the BC group, 21 (39.6%) were premenopausal or perimenopausal and 32 (60.4%) were postmenopausal; in the BBD group, 12 (57.1%) were postmenopausal and 9 (42.9%) were premenopausal or perimenopausal (**Table 1**). Regarding endocrine therapy allocation, AIs were prescribed exclusively to postmenopausal women, consistent with clinical guidelines. TAM was prescribed to premenopausal or perimenopausal women (n=21) and AIs were prescribed to postmenopausal women (n=32). The median follow-up time for the entire study population was 34.6 months (range: 20–41 months). At the 1-year postoperative assessment, thyroid function tests were completed for 66 subjects (89.2%), including 49 BC patients (92.5%) and 17 BBD patients (81.0%). At the 3-year postoperative assessment, a total of 61 subjects (82.4%) completed the evaluation, including 44 BC patients (83.0%) and 17 BBD patients (81.0%) (**Table 1**). Missing data at follow-up visits resulted from patients not returning as scheduled.

**Table 1:** Baseline demographic and clinicopathological characteristics of the study population.

Characteristic	BC patients (n=53)	BBD patients(n=21)	P value
Demographics			
Age (years), mean ± SD	62.62 ± 9.71	56.29 ± 14.41	0.074
BMI (kg/m²), mean ± SD	25.09 ± 2.75	25.77 ± 2.91	0.361
Menopausal status, n (%)			
Premenopausal/ perimenopausal	21(39.6)	9(42.9)	0.790
Postmenopausal	32(60.4)	12(57.1)	
Baseline Thyroid Function			
TSH (μIU/mL)	1.92(1.66,2.96)	1.90(1.65,2.93)	0.946
FT3 (pmol/L)	4.90(4.71,5.22)	4.85(4.70,5.19)	0.627
FT4 (pmol/L)	18.35(16.50,19.62)	17.11(16.03,19.34)	0.101
TgAb (+), n (%)	4(7.5)	2(9.5)	>0.99
TPOAb (+), n (%)	3(5.7)	1(4.8)	>0.99
Thyroid ultrasound: diffuse heterogeneous echotexture, n (%) ^a^	4(7.5)	1(4.8)	>0.99
Tumor Characteristics			
Tumor size (cm), median (range)	1.5(1.0,2.0)	–	–
Histological type			
IDC	47	–	–
ILC	1	–	–
Other special types ^b^	5	–	–
Molecular subtype			
Luminal A	31	–	–
Luminal B	22	–	–
Primary endocrine therapy			
Tamoxifen (TAM)	21	–	–
Aromatase inhibitors (AIs)	32	–	–

Normally distributed variables (age, BMI) are presented as mean ± SD and compared by the independent-samples t-test; non-normally distributed variables (TSH, FT3, FT4) are presented as median (interquartile range) and compared by the Mann–Whitney U test; and categorical variables are presented as frequencies (percentages) and compared by Fisher’s exact test. BC, breast cancer; BBD, benign breast disease; BMI, body mass index; TSH, thyroid-stimulating hormone; FT3, free triiodothyronine; FT4, free thyroxine; TgAb, thyroglobulin antibody; TPOAb, thyroid peroxidase antibody; SD, standard deviation; IQR, interquartile range; IDC: invasive ductal carcinoma; ILC: invasive lobular carcinoma. ^a^ Diffuse heterogeneous echotexture is a common sonographic finding in Hashimoto’s thyroiditis, indicating possible underlying autoimmune thyroid disease; ^b^ Other special types include two invasive papillary carcinomas, one invasive cribriform carcinoma, one invasive mucinous carcinoma, and one invasive tubular carcinoma.

No significant differences were observed between the study groups regarding age, body mass index (BMI), menopausal status, or baseline thyroid function parameters (TSH, FT3, FT4). For thyroid antibodies (TgAb, TPOAb) and ultrasound findings suggestive of Hashimoto’s thyroiditis, the low number of positive events precluded reliable statistical inference, and no significant differences were found (all P > 0.05, with limited power) (**Table 1**).

### Between-group comparison of longitudinal thyroid function changes

In the primary analysis comparing BC patients with BBD controls, GLMM with a Gamma distribution and log link revealed significant time-by-group interactions for all three thyroid parameters (**Table 2**). For TSH, the interaction LRT P was 0.018, with an exponentiated coefficient (Exp[β]) of 1.38 (95% CI: 1.11–1.72) for the 3-year change, indicating a 1.38-fold greater increase in BC patients relative to BBD controls. For FT3, the interaction LRT P was 0.016 (Exp[β] = 0.81 [95% CI: 0.70–0.93]), corresponding to a 19% greater decline in BC patients. For FT4, the interaction LRT P was <0.001 (Exp[β] = 0.78 [95% CI: 0.73–0.83]), representing a 22% greater relative decrease in BC patients. No significant main effects of time or group were observed (all P > 0.05).

**Table 2:** Changes in thyroid function in BC Patients and BBD controls.

Parameter	Group	n (T0/T1/T3)	T0	T1	T3	P-Value	Estimate (β) [95% CI]
TSH (μIU/mL)	BC	53/49/44	1.92(1.66,2.96)	2.37(2.08,3.34)	3.06(2.38,4.23)	0.018*	1.38[1.11-1.72]
	BBD	21/17/17	1.90(1.65,2.93)	2.04(1.63,3.13)	2.55(1.63,3.04)		
FT3 (pmol/L)	BC	53/49/44	4.90(4.71,5.22)	4.70(3.61,4.96)	4.14(2.94,4.65)	0.016*	0.81[0.70-0.93]
	BBD	21/17/17	4.85(4.70,5.19)	4.80(4.58,5.24)	4.70(4.43,5.24)		
FT4 (pmol/L)	BC	53/49/44	18.35(16.50,19.62)	16.34(14.91,17.21)	15.26(13.11,16.21)	<0.001*	0.78[0.73-0.83]
	BBD	21/17/17	17.11(16.03,19.34)	17.90(16.30,19.56)	16.80(15.79,19.45)		

Data are presented as median (interquartile range). Generalized linear mixed models (GLMM) with Gamma distribution and log link, random intercept per subject. Exp(β) is the ratio of geometric mean changes from baseline to 3 years (BC vs. BBD); values >1 indicate a greater increase (or smaller decrease) in the BC group. P-Value from likelihood ratio test comparing models with and without the time-by-group interaction term. No significant main effects of time or group were observed (all P > 0.05). T0, baseline; T1, 1 year post-surgery; T3, 3 years post-surgery.

To assess robustness to potential confounding, we repeated the GLMM analyses separately adjusting for age, BMI, menopausal status, and baseline TSH level. The time-by-group interaction for FT4 remained statistically significant in all models (all P < 0.001). For TSH, interaction LRT P values ranged from 0.004 to 0.020, and for FT3 from 0.014 to 0.016, depending on the covariate. Effect estimates remained consistent with the primary analysis.

### Subgroup analysis by endocrine therapy type

In a subgroup analysis of the 53 breast cancer patients, 21 received TAM and 32 AIs. Baseline characteristics were comparable between the two groups (**Table 1**). As shown in **Table 3**, the TAM group had numerically lower FT4 levels at 3 years than the AIs group. The time-by-treatment interaction for FT4 was statistically significant (LRT P = 0.022; Exp[β] = 0.90, 95% CI: 0.83–0.97), indicating that FT4 declined approximately 10% more in the TAM group over the 3-year follow-up. For TSH (LRT P = 0.508) and FT3 (LRT P = 0.204), the interactions were not significant. These findings suggest a possible association between TAM and a greater FT4 decline, but confounding with menopausal status precludes a causal interpretation.

**Table 3:** Subgroup analysis of thyroid function changes in breast cancer patients by endocrine therapy type.

Parameter	Group	n (T0/T1/T3)	T0	T1	T3	P-value	Exp(β) [95% CI]
TSH (μIU/mL)	AIs	32/31/26	1.82(1.62,2.16)	2.27(1.92,2.98)	2.97(2.32,3.74)	0.508	0.96[0.76-1.21]
	TAM	21/18/18	2.34(1.87,3.62)	3.56(2.36,4.68)	3.44(2.34,5.11)		
FT3 (pmol/L)	AIs	32/31/26	4.90(4.80,5.26)	4.70(3.92,4.90)	4.21(3.07,4.72)	0.204	0.87[0.73-1.03]
	TAM	21/18/18	4.80(4.31,5.15)	4.43(2.45,5.16)	3.67(2.28,4.58)		
FT4 (pmol/L)	AIs	32/31/26	19.00(17.26,19.76)	16.34(15.60,17.60)	15.53(14.15,16.26)	0.022	0.90[0.83-0.97]
	TAM	21/18/18	17.74(16.18,19.46)	15.80(13.02,17.02)	13.90(10.27,15.38)		

Data are presented as median (interquartile range). GLMM with Gamma distribution and log link, random intercept per subject. Exp(β) is the ratio of geometric mean changes from baseline to 3 years (TAM vs. AIs); values <1 indicate a greater decrease (or smaller increase) in the TAM group. P-Value from likelihood ratio test. Only FT4 showed a statistically significant interaction (P = 0.022). T0, baseline; T1, 1 year post-surgery; T3, 3 years post-surgery.

### Subgroup analysis by tumor grade

To explore whether tumor grade modifies the effect of endocrine therapy on thyroid function, we stratified the 53 BC patients into low-grade (Grade I, n=29) and high-grade (Grade II/III, n=24) groups. Longitudinal changes in thyroid parameters are summarized in **Table 4**. Both grade groups showed similar trends of increasing TSH and decreasing FT3 and FT4 over the three-year follow-up. The time-by-grade interaction did not reach statistical significance for TSH (LRT P = 0.210) or for FT3 (LRT P = 0.186). For FT4, the time-by-grade interaction was not statistically significant when assessed jointly across both follow-up time points (LRT P = 0.073). At the 3-year time point alone, however, the estimated ratio of geometric mean change was 1.09 (95% CI: 1.01-1.18; Wald P = 0.026), suggesting a marginally smaller decline in FT4 among higher-grade patients. Given the non-significant overall interaction and the exploratory nature of this subgroup analysis, this 3-year finding should be interpreted with caution.

**Table 4:** Longitudinal changes in thyroid function according to tumor grade in breast cancer patients.

Parameter	Grade	n (T0/T1/T3)	T0	T1	T3	P-Value	Exp(β) [95% CI]
TSH (μIU/mL)	I	29/29/24	1.92(1.67,3.34)	2.44(2.12,3.56)	3.69(2.50,5.04)	0.210	0.84[0.66-1.05]
	II/III	24/20/20	1.93(1.68,2.38)	2.28(2.06,3.11)	2.56(2.14,3.23)		
FT3 (pmol/L)	I	29/29/24	4.90(4.51,5.17)	4.70(3.61,5.08)	3.52(2.75,4.62)	0.186	1.13[0.95-1.34]
	II/III	24/20/20	4.85(4.73,5.32)	4.70(3.43,4.90)	4.23(3.65,4.65)		
FT4 (pmol/L)	I	29/29/24	18.37(17.47,19.57)	16.38(14.80,17.11)	14.26(12.43,16.21)	0.073	1.09[1.01-1.18]
	II/III	24/20/20	18.11(16.20,19.64)	16.16(14.81,17.35)	15.33(14.17,16.25)		

Data are presented as median (interquartile range). Exp(β) is the ratio of geometric mean change from baseline to 3 years (Grade II/III and Grade I), estimated from GLMM with Gamma distribution and log link. P-Value from likelihood ratio test comparing models with and without the time × grade interaction term. T0, baseline; T1, 1 year post-surgery; T3, 3 years post-surgery.

### Prevalence of hypothyroidism

To evaluate whether BC patients receiving endocrine therapy have a higher risk of developing hypothyroidism, we compared the 3-year cumulative incidence of hypothyroidism (including overt and subclinical hypothyroidism) between the BC and BBD groups. The incidence was higher in the BC group than in the BBD group (27.3% vs. 5.9%), but the difference did not reach statistical significance (P = 0.088) (**Table 5**).

**Table 5:** Comparison of hypothyroidism prevalence between BC and BBD patients at 3 years post-operation.

Group	n	Hypothyroidism, n (%)	Subclinical Hypothyroidism, n (%)	Total Hypothyroidism, n (%)	P Value
BC	44	8(18.2)	4(9.1)	12(27.3)	0.088
BBD	17	0(0)	1(5.9)	1(5.9)	

The sample size refers to patients who completed thyroid function assessment at 3 years postoperatively. Overt hypothyroidism defined as TSH > 4.34 μIU/mL with decreased FT4. Subclinical hypothyroidism defined as TSH > 4.34 μIU/mL with normal FT4. Total hypothyroidism includes both overt and subclinical cases. P value calculated using Fisher’s exact test for total hypothyroidism prevalence between groups.

## Discussion

Our findings demonstrated that patients with early-stage luminal breast cancer exhibited significant alterations in thyroid function over a 3-year course of endocrine therapy compared to those with BBD. These alterations were characterized by a marked increase in serum TSH levels, accompanied by significant declines in FT3 and FT4. Notably, although these longitudinal changes were statistically significant, the mean values for all three parameters remained within the normal reference ranges at all time points, indicating that most patients did not develop overt thyroid dysfunction. The differential trajectories between the BC and BBD groups were statistically significant, suggesting a potential influence of either the breast cancer itself or its ensuing endocrine therapy on thyroid homeostasis.

In the endocrine therapy type subgroup analysis, we observed a statistically significant time-by-treatment interaction for FT4 (P = 0.022), with FT4 declining approximately 10% more in the TAM group than in the AIs group over three years. No significant differences were found for TSH or FT3. While a systematic review by Marina et al. concluded that TAM is associated with mild subclinical hypothyroidism, primarily reflected by elevated TSH and total T4, with inconsistent effects on free thyroid hormones (10), the interpretation of our FT4 finding is substantially limited by the collinearity between treatment type and menopausal status in our cohort. Therefore, the greater FT4 decline observed in the TAM group cannot be attributed solely to TAM.

Both the breast and thyroid are endocrine organs regulated by the hypothalamic-pituitary axis, and their hormonal interaction has been substantiated by multiple studies (15–17). Mammary tissue is not only responsive to thyroid hormones but also exhibits sensitivity to sex steroids like estrogen, establishing a biological basis for hormonal cross-talk (18). The thyroid suppression observed in our BC cohort following endocrine therapy may partially stem from a disruption of this delicate equilibrium.

At the molecular level, thyroid hormones exert their effects primarily through nuclear receptors (TRα and TRβ), which are widely expressed in breast cancer cells and interact closely with estrogen receptor (ER) signaling pathways (8, 19). For example, Hall et al. demonstrated *in vitro* that T3 promotes the proliferation of ER-positive breast cancer cells in a dose-dependent manner, an effect that is inhibited by ER antagonists, suggesting that T3’s mitogenic action is at least partly mediated via ER signaling (8). Conversely, Lopez-Mateo et al. found that in TRβ-expressing MCF-7 cells, T3 significantly inhibited breast cancer stem cell self-renewal and downregulated ERα (20). Wahdan-Alaswad et al. further showed that combined T3 and estradiol treatment accelerated tumor growth and induced TAM resistance, highlighting a direct molecular link between thyroid hormone excess and endocrine therapy efficacy (21).

Beyond nuclear receptor pathways, thyroid hormones can also activate non-genomic signaling. Tawfik et al. reported that T3 enhances mitochondrial calcium transfer and oxidative phosphorylation via upregulation of inositol trisphosphate receptor type 3, providing energetic support for breast cancer cell proliferation in an ER-dependent manner (22). In this context, the pattern of elevated TSH and decreased FT3/FT4 observed in our BC patients could be interpreted as a compensatory feedback mechanism: the body may attempt to attenuate potential tumor-promoting signals by reducing circulating thyroid hormone levels during anti-estrogen therapy.

We also explored whether tumor grade, as a proxy for disease severity, influenced the observed changes. Subgroup analysis comparing patients with grade II/III versus grade I malignancy revealed no statistically significant time-by-grade interactions for TSH (P = 0.210) or FT3 (P = 0.186), and only a borderline interaction for FT4 (P = 0.073). These findings suggest that the degree of tumor differentiation did not substantially modify the trajectory of endocrine therapy-related thyroid dysfunction. This is particularly relevant in the context of nonthyroidal illness syndrome (NTIS), which typically manifests as decreased FT3 with normal or low TSH in patients with advanced illness. If NTIS were a major driver, one would expect a more pronounced FT3 reduction in the higher-grade subgroup. The absence of such a difference does not support NTIS as a major contributor to our findings, suggesting that the alterations are more likely attributable to the endocrine therapy itself.

Consistent with Jha et al., our patients exhibited significant post-treatment thyroid function alterations, characterized by elevated TSH and decreased FT3 and FT4 levels. Although the Jha et al. cohort received chemotherapy, their postoperative thyroid function trends resembled those in our endocrine therapy cohort, suggesting that endocrine therapy per se may be a key driver of these changes (23). Previous research has demonstrated that chemotherapy can significantly impact thyroid function in breast cancer patients (14, 24, 25). Furthermore, a systematic review highlighted a significant risk of hypothyroidism following radiotherapy to the supraclavicular region in breast cancer patients (26), and a prospective study by Farshchian et al. found that 9.5% of patients developed radiation-induced hypothyroidism six months post-treatment (27). By excluding patients who underwent these treatments, our study offers a clearer assessment of the independent impact of endocrine therapy itself on thyroid function.

Despite the statistical significance of these findings, the observed mean values of TSH, FT3, and FT4 remained largely within normal reference ranges, and the overall incidence of hypothyroidism did not differ significantly between groups. These results suggest that while endocrine therapy may induce measurable shifts in thyroid function, these changes are unlikely to be clinically meaningful in most patients.

Importantly, the study has several limitations. The stringent exclusion criteria, while necessary to reduce confounding, resulted in a small sample size and may have introduced selection bias. Only 53 of 2,366 screened breast cancer patients were included, which limits the generalizability of the findings. We did not measure TBG, reverse T3, or thyrotropin receptor antibodies, which limits our mechanistic understanding and the characterization of autoimmune thyroid status. Furthermore, treatment type was completely confounded with menopausal status, preventing any independent assessment of treatment-specific effects. Therefore, the observed differences in FT4 between TAM and AIs should be interpreted with caution.

In conclusion, while adjuvant endocrine therapy for early luminal breast cancer may be associated with subclinical alterations in thyroid function over three years, these changes do not appear to be clinically significant based on the current data. Thyroid function monitoring may be considered in selected patients receiving long-term endocrine therapy, but routine monitoring is not yet justified. Future prospective studies with larger sample sizes, comprehensive thyroid panels, and adjustment for menopausal status are needed to clarify the mechanisms and clinical implications of these findings.

## Data Availability

The raw data supporting the conclusions of this article will be made available by the authors, without undue reservation.
